# Propagation of Bacteria

**Published:** 1883-06

**Authors:** 


PROPAGATION OF BACTERIA.—The investigations of Darwin and Pasteur have shown that earthworms are an important vehicle of transportation of minute organisms. Professor Schnetzler has lately demonstrated that a certain number of living bacteria and their germs are always found in the interior of earthworms. It is true that bacteria in enormous quantities are floating in the air, and we have a small apparatus at our disposal by which we can easily convince ourselves of this. This is none other than the nasal cavity, which retains those minute air-particles upon its mucous membrane. To observe these we must syringe the nose with distilled water, and collect the obtained fluid in a perfectly clean watch glass. Magnified 700 to 800 times, we find among various other particles living bacteria. If we keep this fluid hermetically closed, we find that after a few days these bacteria have multiplied largely, and bacterium termo, vibrio, spirillum, bacillus subtilis, with sometimes even a few infusoria, are present. Prof. Schnetzler has successfully cultivated these germs in a solution of gelatine and distilled water. The question is, why do not these bacteria multiply in the nasal cavity and find their way into the trachea and lungs? Their advance is undoubtedly stopped by the vibratory movements of the cilia, and by the slight alkaline reaction of the nasal cavity. Colin has demonstrated that bacteria which produce an acid fermentation, will die in fluids of alkaline reaction. Contagious bacteria will multiply on mucous membranes at a wonderful rate, as shown in the micrococci of diphtheria, which obtain entrance into the respiratory organs through the atmosphere, as also the bacteria of anthrax. Bacillus tuberculosis, as stated by Koch, can be carried from one person to another by means of the respiratory passages. Prof. Schnetzler beiieves that hay fever originates in the same way, by bacteria, which are taken up by the nasal cavity. While the development of bacteria on normal mucous membranes is generally limited, millions are found in the excrements of healthy children.—*Monatsschrift des Vereins Deutscher Zahnkuenstler.*

